# Recrudescence of Scarlet Fever and Its Implications for Dental Professionals

**DOI:** 10.1016/j.identj.2023.03.009

**Published:** 2023-04-14

**Authors:** Victor Haruo Matsubara, Janina Christoforou, Lakshman Samaranayake

**Affiliations:** a Dental School, University of Western Australia, Perth, Western Australia, Australia; b Faculty of Dentistry, The University of Hong Kong, Hong Kong, Special Administrative Region, China; c Hamdan Bin Mohammed College of Dental Medicine, Dubai, United Arab Emirates

**Keywords:** Scarlet fever, Resurgence, Dentistry, Implications

## Abstract

A significant increase in the incidence of scarlet fever, mainly in Europe, has been noted during the COVID-19 postpandemic period. Scarlet fever is caused by a pyrogenic exotoxin-producing streptococcus—*Streptococcus pyogenes*—responsible for more than 500,000 deaths annually worldwide. Superantigens (SAgs) secreted by this Group A streptococcus (GAS) usually overstimulate the human immune system, causing an amplified hypersensitivity reaction leading to initial symptoms such as sore throat, high fever, and a sandpaper-like skin rash. There could be concurrent oral manifestations known as “strawberry tongue” or “raspberry tongue,” which may be first noted by oral health professionals. The early diagnosis and treatment of this disease is critical to obviate the development of local and systemic sequelae such as acute rheumatic fever, endocarditis, and glomerulonephritis. Antibiotics should be prescribed early to mitigate its duration, sequelae, and community spread. Dental practitioners should be aware of the early symptoms of scarlet fever for infection detection, emergency patient management, and appropriate referral. This concise review outlines the prevalence, pathogenicity, oral and systemic manifestations, as well as the dental implications of scarlet fever.

## Introduction

Scarlet fever, caused by *Streptococcus pyogenes*, was considered a fatal disease during the 19th and early 20th centuries. However, the morbidity and mortality of the disease was markedly reduced worldwide when antimicrobials such as penicillin became widely available. The disease—sometimes referred to as “scarlatina”—is an acute, contagious infection caused by toxins secreted by *Streptococcus pyogenes* belonging to Lancefield Group A streptococci (GAS).^1^ It is an important human pathogen, exclusively adapted to the human body, and its major human habitat is saliva and the pharynx.^2^^,^^3^ GAS infections, in general, account for more than 500,000 deaths annually worldwide.^4^ Other life-threatening infections due to GAS include pneumonia, bacteraemia, necrotising fasciitis, myonecrosis, and streptococcal toxic shock syndrome (STSS).^5^, ^6^, ^7^, ^8^

Historically, scarlet fever was considered a benign infection of early childhood, but its status changed in the early 19th century, when case-fatality rates dramatically started to surpass 15%.^9^ Despite being almost eradicated in the 20th century, due to the advent of antibiotics and appropriate disease management, epidemics of scarlet fever were reported in North East Asia^10^ and the United Kingdom^11^ in 2011 and 2014, respectively. GAS outbreaks were also reported in other parts of the world before the COVID-19 pandemic.^12^, ^13^, ^14^ A more recent upsurge of scarlet fever and invasive GAS infection amongst children younger than 10 years in a number of European countries, including France, the Netherlands, Sweden, Ireland, and the UK, particularly since September 2022, has rekindled the attention given to this disease.^15^

The latest data show a substantial increase in the number of cases and deaths associated with GAS infections. The UK Health Security Agency (UKHSA) reported 27,486 confirmed scarlet fever cases and 94 deaths from September 2022 to December 2022, across all age groups in England, including 21 individuals younger than 18 years. This compares with a total of 3287 infections in the same period during the last comparably high season in 2017–2018.^16^ The potential causes of these recent outbreaks of scarlet fever remain unclear. According to the UKHSA, the social distancing measures implemented during the coronavirus pandemic may have interrupted the disease cycle.^16^ Furthermore, it has been surmised that the lack of exposure of young children to GAS during COVID-19 lockdowns may have weakened their immune response against the bacteria.^17^

Studies on scarlet fever outbreaks prior to the COVID-19 pandemic show epidemiologic evidence and molecular analysis supporting that interstrain genetic variability and antibiotic resistance may have significantly influenced the evolution and virulence of GAS strains associated with scarlet fever, particularly in North-East Asia.^18^^,^^19^ In contrast, there is no evidence to date of new GAS strains or increased antibiotic resistance associated with the recent epidemic in the UK.^16^

## Transmission and pathophysiology

Streptococcal pyrogenic exotoxins secreted by GAS, also known as erythrogenic toxins, are considered to be the major agents driving the pathophysiology of scarlet fever. These toxins induce inflammation by nonspecifically activating T cells and stimulating the production of inflammatory cytokines of the immune system.^3^^,^^20^ In addition, previous exposure to *S pyogenes* likely contributes to an amplified hypersensitivity reaction with cytokine release and leukocyte infiltration, which is further accentuated by bacterial superantigens (SAgs)^20^ that are powerful immunostimulatory bacterial proteins. At least 14 genetically distinct SAgs have been reported,^21^ and each *S pyogenes* strain typically encodes different repertoires of SAgs.^1^ Specific exotoxins of GAS can also cause autoimmune diseases and reactions, and *S pyogenes* infections in some may lead to rheumatic fever and glomerulonephritis.^22^

The primary habitat of GAS in humans is the oropharynx, but they can rarely colonise the skin, invade the epithelium, and cause invasive disease.^23^ However, GAS are not always associated with disease. One study noted that approximately 12% of children of all ages are “silent” carriers of the organism, which is associated with asymptomatic paediatric streptococcal pharyngitis.^24^

*S pyogenes* infections are highly contagious. Transmission occurs by close contact with an infected person and through droplet spread via coughs or sneezes. Direct contact with the saliva or nasal fluids of infected individuals, skin contact with infected lesions, and direct contact with objects or surfaces contaminated with GAS (fomites) are other routes of spread.^25^ Foodborne transmission is uncommon though sometimes associated with outbreaks in the past.^26^ The likelihood of disease transmission is relatively high in the acute symptomatic stage of the infection due to profuse colonisation of the upper respiratory tract by GAS.^23^

## Clinical features: signs and symptoms

**Fig. 1:**
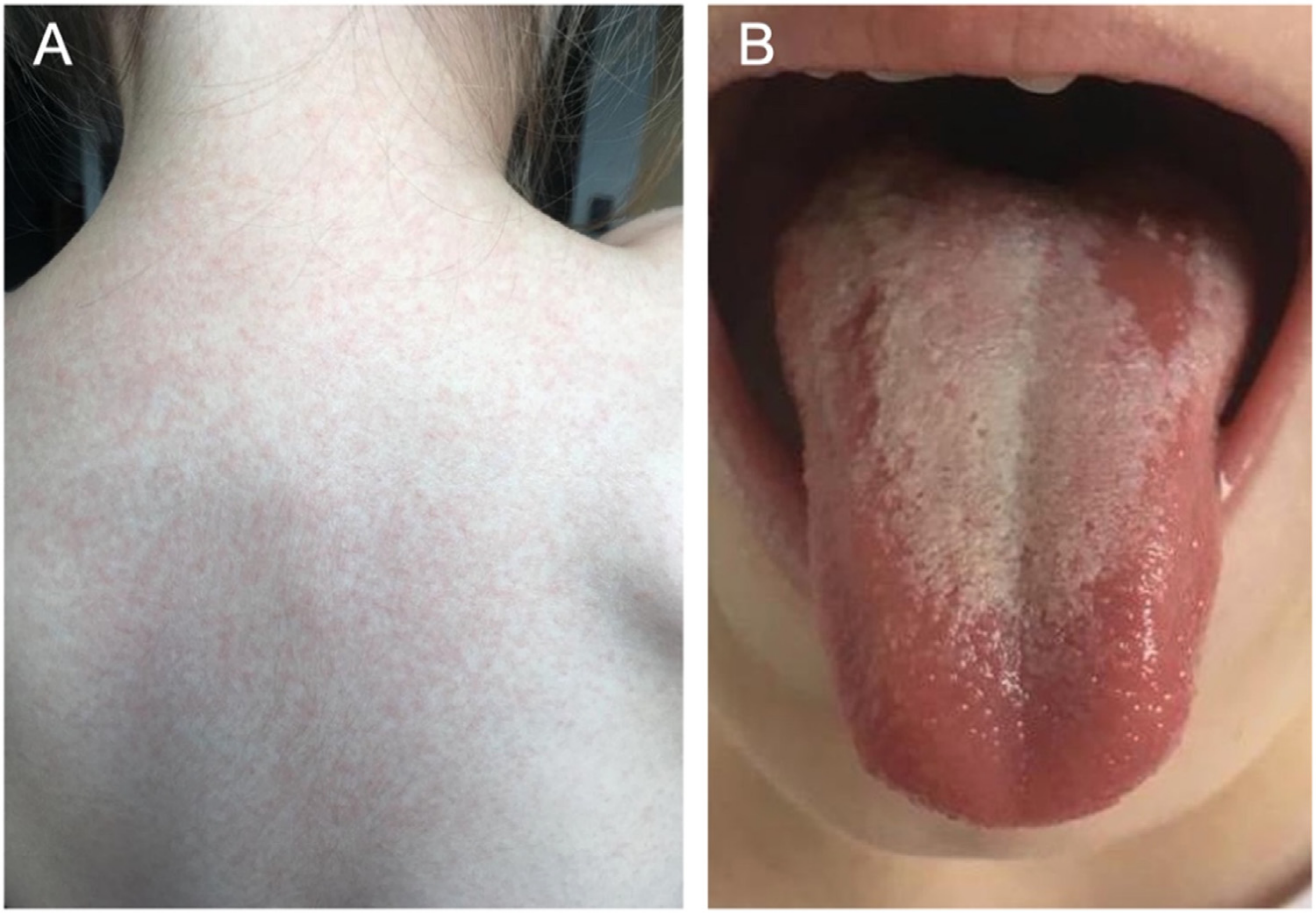
Sandpaper-like skin rash on the back (A) and strawberry-like appearance of the tongue (particularly at the anterior region) (B) seen in a 4-year-old patient with scarlet fever. (Images kindly provided by Dr Zuzanna Ślebioda.)^30^

**Fig. 2:**
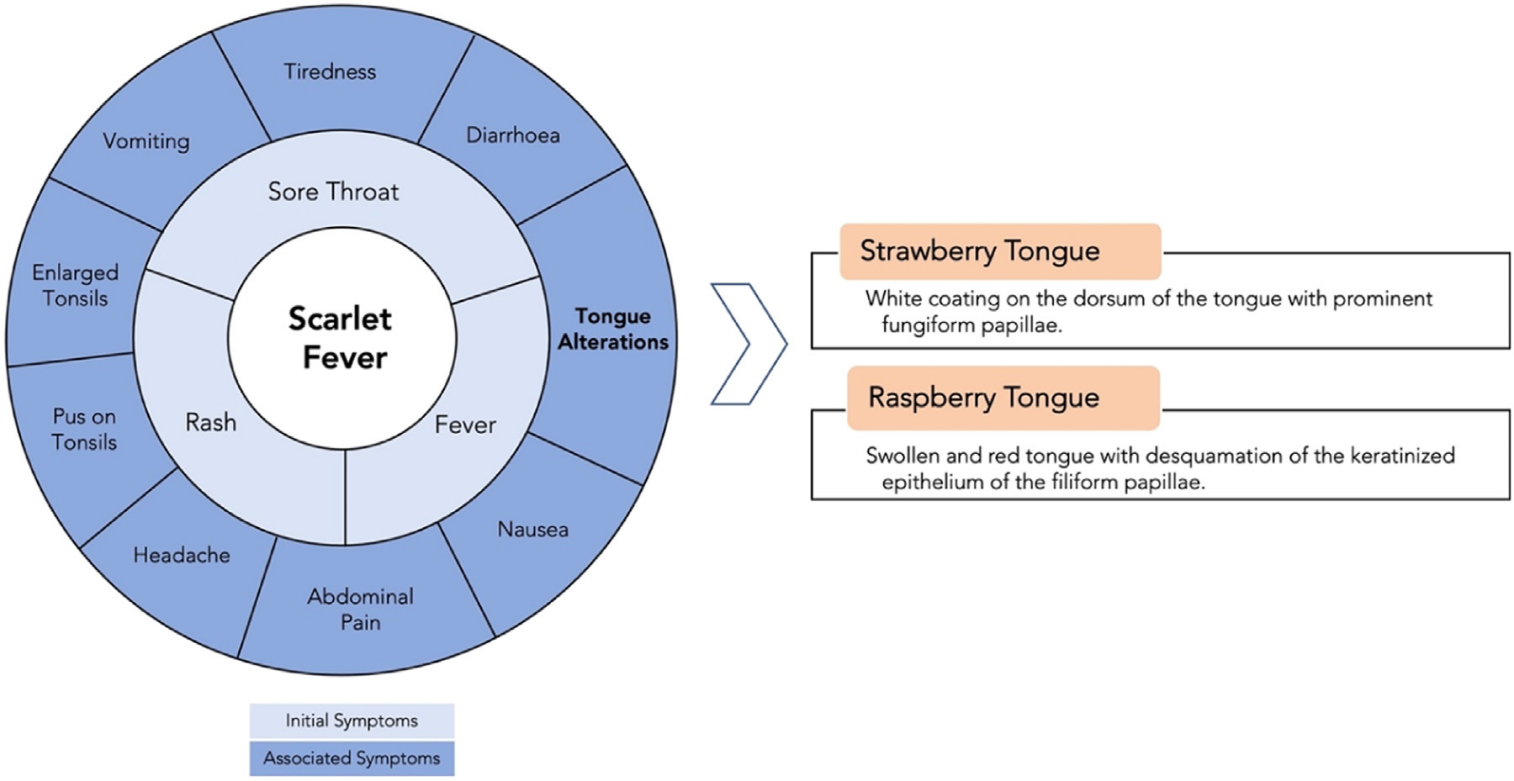
Clinical features of scarlet fever.

Scarlet fever can affect people of any age, but it typically seen in school-aged children and young adults between 5 and 15 years, particularly during winter and spring.^2^^,^^26^ The disease is characterised by a sore throat that is erythematous and oedematous, high fever, and a sandpaper-like skin rash that can be itchy (Figure 1A). Fever and sore throat are usually the first symptoms to be noted followed by rash and tiredness.^27^ Abdominal pain, vomiting, dry mucous membranes, and tender anterior cervical lymphadenopathy are also common^28^ (Figure 2).

The disease, after 2 to 3 days, progresses from the maculopapular exanthematous skin rash to an acute pharyngitis. The rash is centrifugal, usually beginning in the axillary and inguinal region and extending around the neck and the back before spreading to other parts of the body.^29^ The skin rash in scarlet fever is characterised by the appearance of the so-called Pastia's lines, due to accentuation of the red rash in flexor creases under the arms and the groin. After around 7 to 10 days, the rash reaches the extremities, palms, and soles, followed by desquamation of the skin in these regions.^28^

In addition to scarlet fever, GAS can also cause more serious diseases due to invasive infections and systemic spread, especially in immunocompromised patients. These include meningitis; STSS, which may cause organs to fail; and necrotising fasciitis. The latter, also known as the “flesh-eating disease,” affects the tissues under the skin.^5^

## Orofacial manifestations

There are both extraoral and intraoral manifestations associated with scarlet fever. A characteristic, virtually pathognomonic, facial sign that appears concurrently with the initial skin rash is a flushed face with a circumoral pallor (ie, lighter skin around the mouth relative to the other flushed areas of the face).

Intraorally, a yellowish white coating with red papillae may initially cover the tongue, with the subsequent fading of the coating leaving a residual red tongue termed the “strawberry tongue” or “raspberry tongue” (Figure 1B).^30^ The prominence of the filiform and fungiform papillae due to the associated swelling and redness leads to this appearance. As the infection progresses, the dorsum of the tongue becomes swollen and erythematous as a result of the desquamation of the keratinised epithelium of the papillae and the associated inflammation.^31^ Fissured lips and enlarged oedematous tonsils with exudates are also common oral signs of the infection.

## Differential diagnosis

Viral infections, such as measles and rubella, present with rashes that resemble those of scarlet fever with subsequent skin desquamation a few days later. The primary, almost pathognomonic, characteristic that differentiates scarlet fever from measles and rubella is the presence of Pastia's lines with persistent erythema that does not disappear when the affected skin area is compressed. Further, there are no signs of upper respiratory inflammation in scarlet fever.^29^ Epstein-Barr virus and adenovirus infections occur commonly in the same age group, present with similar symptoms, and can be misdiagnosed as scarlet fever.^28^ The pharyngeal manifestation of GAS infections may be similar to those of diphtheria and pseudomembranous candidosis, although in the latter case a distinct false white membrane is evident.

When rash and abdominal pain are present, scarlet fever can be mistaken for typhoid fever, cholesterol emboli syndrome, or systemic lupus erythema. However, if the symptoms include adenopathy associated with the rash, the differential diagnosis can also include Kawasaki disease.^29^

A diagnosis of scarlet fever relies mainly on accurate medical history and a thorough clinical examination of lesions. Its diagnosis is challenging in the early stages of the disease due to the great variation in severity of signs and symptoms. Hence, a tonsillar swab for culture can be beneficial for confirmation purposes.^32^ In the laboratory, it can be quickly diagnosed by rapid antigen tests (rapid strep) or bacterial culture.

## Management

Management of scarlet fever is based on reducing symptoms and preventing complications whilst controlling the risks of transmission.^27^ Once a diagnosis has been made, patient isolation, good hand hygiene, and respiratory etiquette are important to reduce transmission. Antibiotics should be prescribed early, as this will decrease the duration of the infection, reduce the risk of its spread, and also mitigate the development of complications.

Penicillin group drugs are the antibiotic of choice, and a number of other drugs such as cephalexin, cefadroxil, and clarithromycin could be given for penicillin-allergic patients.^33^ The drug dosage and duration of these are provided in Table 1.

**Table 1:** Antibiotic regimens recommended for scarlet fever.

Drug, route	Dose or dosage	Duration or quantity
**For individuals without penicillin allergy**		
Penicillin V, oral	Children: 250 mg twice daily or 3 times daily; adolescents and adults: 250 mg 4 times daily or 500 mg twice daily	10 d
Amoxicillin, oral	50 mg/kg once daily (max = 1000 mg); alternate: 25 mg/kg (max = 500 mg) twice daily	10 d
Benzathine penicillin G, intramuscular	<27 kg: 600,000 U; ≥27 kg: 1,200,000 U	1 dose
**For individuals with penicillin allergy**		
Cephalexin, oral	20 mg/kg/dose twice daily (max = 500 mg/dose)	10 d
Cefadroxil, oral^a^	30 mg/kg once daily (max = 1 g)	10 d
Clindamycin, oral	7 mg/kg/dose 3 times daily (max = 300 mg/dose)	10 d
Azithromycin, oral	12 mg/kg once daily (max = 500 mg)	5 d
Clarithromycin, oral	7.5 mg/kg/dose twice daily (max = 250 mg/dose)	10 d

a Avoid in individuals with immediate-type hypersensitivity to penicillin. Modified from Shulman et al.^32^

Additionally, the administration of paracetamol will help reduce fever and pain. Individuals affected should maintain proper nutrition and reduce the risk of dehydration by copious liquid intake.

Early diagnosis and treatment of scarlet fever are important so as to avoid both local and systemic complications. Local complications may present as either suppurative sequalae or nonsuppurative sequelae. Suppurative complications result from local or haematogenous spread of the organism and include peritonsillar abscesses, retropharyngeal abscess, cervical lymphadenitis, and invasive GAS disease. Hepatitis, gallbladder hydrops, or splenomegaly have been rarely reported in a few patients. Nonsuppurative sequelae include poststreptococcal glomerulonephritis and rheumatic carditis. These complications occur after the original infection resolves and involve sites distant to the initial GAS infection site. They are considered to be the result of the immune response and not of direct GAS infection.

## Considerations for dental professionals

Due to the characteristic oral presentation of this disease, dental professionals must be aware of its signs and symptoms for early recognition and diagnosis and appropriate referral as well as immediate symptomatic management. Patients with suspected cases should be advised on the importance of infection control including wearing of face masks so as to minimise the spread of the infection.

As dental professionals currently adhere to standard infection control measures coupled with transmission-based precautions, particularly due to the COVID-19 endemicity, there should be no additional risk of scarlet fever transmission of the disease in the dental clinics.^34^ Nevertheless, it is salutary to have a strategic plan to manage scarlet fever amongst patients, some features of which are annotated below:

• The dental team should be alert to peaks and occurrence of scarlet fever outbreaks in the region and maintain an appropriate index of suspicion.
• A management plan for patients with suspected cases should be available and displayed prominently in the clinic, particularly during periods of outbreaks.
• Such a plan should include immediate management and referral data to the appropriate local medical centre dealing with such infectious diseases.
• All elective surgical procedures should be deferred until the patient fully recovers from scarlet fever.
• The local public health protection team and/or the patient's general medical practitioner should be approached for advice on the immediate management aspects of patients with acute cases.
• Additional droplet precautions such as wearing FFP3 respiratory masks may be necessary when acute patients are treated.
• In addition, the patient should be treated at the end of a clinical session, if possible, and a thorough disinfectant of the surgery undertaken thereafter.
• Equipment within the surgery should be kept to a minimum as much as possible during the surgical procedure.

## Conclusions

The recent resurgence in the scarlet fever cases during the postpandemic period is disconcerting. The disease can lead to more severe and life-threatening conditions due to poor management during the initial course of the disease. Oral health professionals, especially dentists, are well placed to recognise the disease and mitigate its spread, as they may be the first to notice the early orofacial signs of the infection. They should be aware of the signs and symptoms of this resurgent infection and also maintain a high degree of vigilance when assessing patients, particularly children. Early detection of probable cases and appropriate management, as outlined above, will prevent not only further disease spread but also lifelong debilitating sequalae for the affected patient.

## Author contributions

VHM collected the information, contributed data, performed the analysis, wrote the paper, revised the paper, and edited the revised paper. JC contributed data, performed the analysis, edited the paper, and edited the revised paper. LS conceived and designed the review, collected the data, contributed data or analysis tools, performed the analysis, wrote the paper, revised the paper, and edited the revised paper.

## Conflict of interest

None disclosed.
